# Nasal syringocystadenoma papilliferum in an elderly subject aggravated by Koebner's phenomenon after surgery: A case report

**DOI:** 10.1016/j.amsu.2021.102678

**Published:** 2021-08-11

**Authors:** Ulrich Opoko, Iro Salissou, Rkia Ajaaouani, Ayoub Sabr, Mohamed Raiteb, Sanaa Elmrini, Soumiya Chiheb, Faiçal Slimani

**Affiliations:** a Faculty of Medicine and Pharmacy, Hassan II University of Casablanca, Casablanca, Morocco; b Department of Stomatology and Maxillofacial Surgery, CHU Ibn Rochd, Casablanca, Morocco; c Department of Dermatology and Venereology, CHU Ibn Rochd, Casablanca, Morocco

**Keywords:** Syringocystadenoma papilliferum, Diagnosis, Surgery, Koebner's phenomenon

## Abstract

Syringocystadenoma papilliferum is a rare benign adnexal tumour of the sweat glands. It is considered an infantile tumour since it preferentially affects the newborn in 50% of cases and the child before puberty in 15–30% of cases. And its preferential location is the head and neck, but rare in the face. And the first line treatment remains surgery. We report here a case of Syringocystadenoma papilliferum in a nasal location in a 70 year old subject with a history of pemphigus vulgaris, treated by surgical excision, whose postoperative course was aggravated by Koebner phenomenon.

## Highlights

• Syringocystadenoma papilliferum is a rare benign adnexal tumour of the sweat glands, with preferential localisation in the head and neck.
• It is often present at birth and in early childhood before puberty, and rare in adults.
• The recommended management is surgery, but certain comorbidities may be responsible for Koebner's phenomenon.
• Koebner's phenomenon is the appearance of typical new skin lesions on wounded areas of otherwise healthy skin.

## 1 Introduction

Syringocystadenoma papilliferum (SCAP) is a rare benign adnexal tumour of the apocrine or eccrine sweat glands [1,2]. In almost 50% of cases, it is present at birth, but in about 15% of cases it appears in early childhood. It develops either de novo or in the majority of cases from a sebaceous hamartom [3,4]. Koebner's phenomenon (KP) is defined as the appearance of typical new skin lesions on wounded areas of otherwise healthy skin. We report here a case of SCAP in the nose of an elderly subject treated by surgical excision with delayed healing.

This article has been reported in with the SCARE criteria [5].

## 2 Case report

**Fig. 1:**
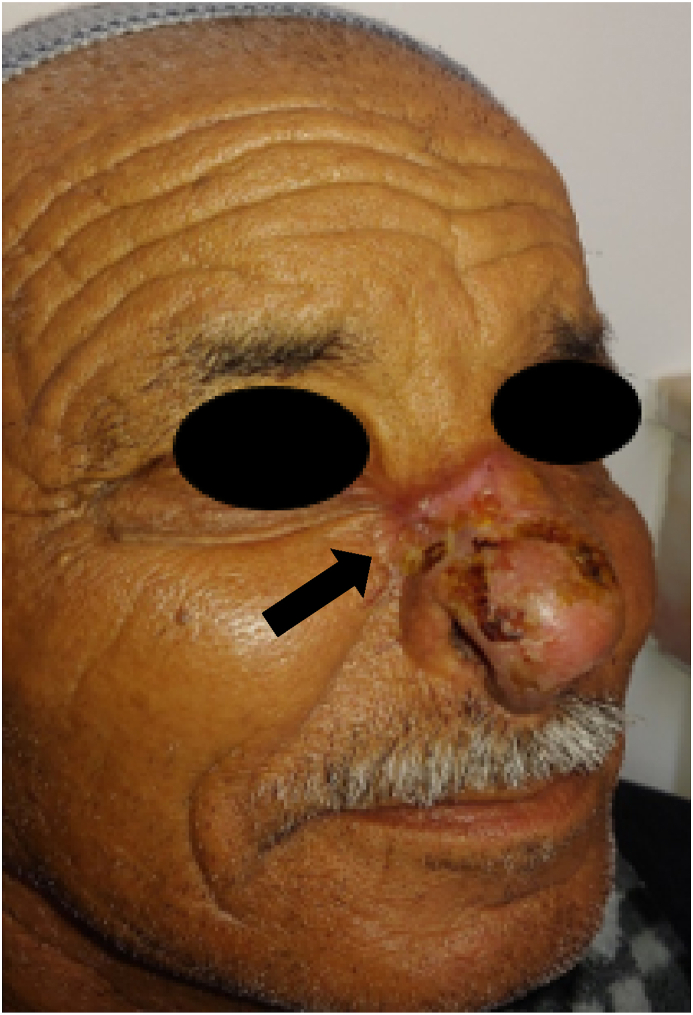
Picture of the patient showing the lesion of the nasal pyramid.

**Fig. 2:**
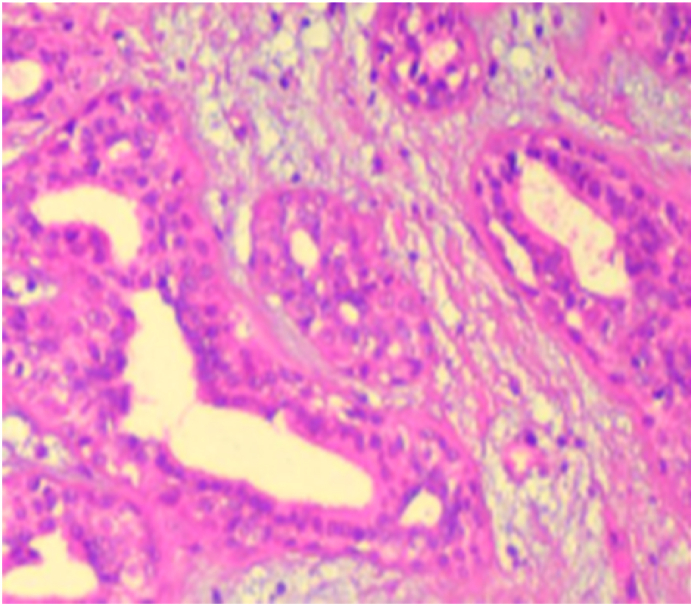
Histological image confirming the diagnosis of papilliferous syringocystadenoma.

A 70 year old patient, followed for 3 years in dermatology for pemphigus vulgaris for which he was in remission under corticotherapy at a dose of 10mg/day and Azathioprine at a dose of 100mg/day. No other particular pathological history was noted. He presented to our department of maxillofacial surgery in Casablanca, for a skin lesion of the nasal pyramid evolving for 02 months, gradually increasing in size. The physical examination revealed a lesion of about 2 cm, on the right side of the nasal pyramid reaching the contact of the right internal canthus, male limited, ulcerated in the centre with a brownish aspect at the bottom, and verrucous in the periphery (Fig. 1). A biopsy was performed under local anesthesia for histological study, showing a proliferation of glandular papillary architecture, with pseudo-cystic invaginations presenting papillary projections, without infiltration of the dermis, making the diagnosis of a papilliferous syringocystic adnexal tumour (Fig. 2). The patient subsequently underwent excision of the lesion, under general anesthesia, with sufficient margins of 5mm confirming the diagnosis.

**Fig. 3:**
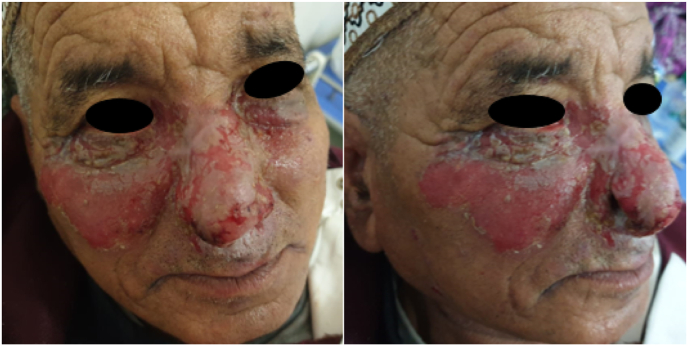
Pictures of the patient at three months post-op showing the appearance of Koebner's phenomenon.

During the post-operative follow-up, there was a lack of healing of the surgical wound after 3 months after surgery, the patient retained an erosion of the surgical wound and an ectropion, with the appearance of ulcerative-bullous lesions over the entire nasal pyramid, extending to the palpebral-jugal regions bilaterally (Fig. 3), and even over the rest of the body at the level of the trunk and the limbs.

The patient was then referred to dermatology for further treatment, where he received local care based on oily dressings, corticosteroid therapy (prednisolone 40 mg/day), immunosuppressant (azathioprine 100 mg/day) and an antiviral (valacyclovir 1g/day) for 10 days because of a herpetic superinfection observed. The evolution over 6 months was favourable, with healing of the lesions and no signs of local recurrence.

## 3 Discussion

Syringocystadenoma papilliferum, also known as intracanalicular adenocystoma, fistulo-vegetative verrucous hidradenoma or papilliferous syringoadenomatous nevus, is a rare adnexal tumour [6]. SCAP occurs preferentially in infancy, with 50% of tumours present at birth, and 15–30% developing before puberty [3,4,6,7]. Our report is an exception to the rule, as our patient was 70 years old.

The most frequent site of localisation is the head and neck. Less frequent sites are the face, chest, abdomen, arms, thighs and perineum [8]. However, in our report, the location was nasal.

It develops either de novo or in the majority of cases from a sebaceous hamartoma. It originates from the appendages of the sweat glands [4]. However, the exact origin is still debated; for some authors it derives from apocrine glands and for others it is of eccrine or mixed eccrine and apocrine origin [4].

The clinical picture is non-specific and often misleading [7,9]. It may present as a solitary erythematous and alopecic plaque that progressively increases in size during adolescence to become nodular, verrucous and papillomatous, or as a solitary nodule or as multiple umbilicated greyish-brown papules [4]. It can vary in size from a few millimetres to several centimetres [4].

The diagnosis is histological [1,4,6]. SCAP is an epithelial proliferation connected to the epidermis and formed by tubular and papillary structures, bordered by a double layer of epithelial cells: the innermost being cylindrical, and the outermost cubic. The tumour stroma is predominantly plasma cells [4,7].

Associations with other tumours, both benign (apocrine cystadenoma, apocrine hydrocystoma, papilliferous hidradenoma and follicular poroma) and malignant (basal cell, verrucous and sebaceous carcinomas) have been reported [6]. However, malignant degeneration to papilliferous syringocystadenocarcinoma is exceptional but possible [4,6,7].

The best treatment is surgical removal. CO2 laser is an alternative treatment when surgery is impossible [3,4,[6], [7], [8]].

Being a benign tumour, the prognosis is generally good, characterised by complete remission with no tumour recurrence and good healing after complete removal. However, in our case, the patient after surgical excision, after three months of surgery and local care of the excision site, was found to have no healing with the appearance at this level and locally (cheeks and eyelids), of extensive ulcerative-bullous lesions preventing healing of the surgical site, then progressively worsening and becoming erosive in places with remote appearances on the trunk and limbs. This finding would correlate with the Koebner phenomenon which is observed in patients followed for pemphigus vulgaris; this is the case in our report where the subject had been followed for pemphigus vulgaris for 3 years.

In the literature, reports of Koebner's phenomenon in pemphigus vulgaris have included stimulation of pemphigus vulgaris lesions by ionising radiation, surgical procedures, burns and chemical peels [10], in our case, surgical trauma is the stimulating factor.

## 4 Conclusion

Syringocystadenoma papilliferum is a rare benign adnexal tumour of the sweat glands, with its non-specific clinical manifestations, histology remains the key to diagnostic confirmation. Although the prognosis is favourable, the recommended management is surgery to avoid possible malignant degeneration or recurrence, but some comorbidities may be responsible for a Koebner phenomenon as in our case, making healing difficult despite complete removal of the tumour.

## Patients concent

Written informed consent was obtained from the parents of the minor girl and the second patient for the publication of this case report and accompanying images. A copy of the written consents is available upon request for review by the editor of this journal.

## Sources of funding

None.

## Provenance and peer review

Not comissioned, externaly peer reviewed.

## Autors contribution

Corresponding author, and writing the paper: Ulrich Opoko; Writing the paper: Iro Salissou, Rkia Ajaaouani, Ayoub Sabr, Raiteb Mohamed, Sanaa Elmrini; Correction of the paper: Soumiya Chiheb,Faiçal Slimani.

## Ethical approval

Our study is exempted of ethical approval.

## Consent

Written informed consent was obtained from the patient for publication of this case report and accompanying images. A copy of the written consent is available for review by the Editor-in-Chief of this journal on request.

## Guarantor

OPOKO ULRICH.

## Declaration of competing interest

Authors of this article have no conflict or computing interest.
